# CXCL17 Expression by Tumor Cells Recruits CD11b^+^Gr1^high^F4/80^−^ Cells and Promotes Tumor Progression

**DOI:** 10.1371/journal.pone.0044080

**Published:** 2012-08-29

**Authors:** Aya Matsui, Hideaki Yokoo, Yoichi Negishi, Yoko Endo-Takahashi, Nicole A. L. Chun, Ichiro Kadouchi, Ryo Suzuki, Kazuo Maruyama, Yukihiko Aramaki, Kentaro Semba, Eiji Kobayashi, Masafumi Takahashi, Takashi Murakami

**Affiliations:** 1 Laboratory of Tumor Biology, Takasaki University of Health and Welfare, Takasaki, Gunma, Japan; 2 Division of Bioimaging Sciences, Center for Molecular Medicine, Jichi Medical University, Shimotsuke, Tochigi, Japan; 3 Department of Human Pathology, Gunma University Graduate School of Medicine, Maebashi, Gunma, Japan; 4 Department of Drug and Gene Delivery Systems, School of Pharmacy, Tokyo University of Pharmacy and Life Sciences, Tokyo, Japan; 5 Department of Biopharmaceutics, School of Pharmaceutical Sciences, Teikyo University, Sagamihara, Kanagawa, Japan; 6 Department of Life Science and Medical Bio-Science, Waseda University, Wakamatsu, Shinjuku, Tokyo, Japan; 7 Division of Development for Advanced Medical Technology, Jichi Medical University, Shimotsuke, Tochigi, Japan; Children’s Hospital Boston & Harvard Medical School, United States of America

## Abstract

**Background:**

Chemokines are involved in multiple aspects of pathogenesis and cellular trafficking in tumorigenesis. In this study, we report that the latest member of the C-X-C-type chemokines, CXCL17 (DMC/VCC-1), recruits immature myeloid-derived cells and enhances early tumor progression.

**Methodology/Principal Findings:**

CXCL17 was preferentially expressed in some aggressive types of gastrointestinal, breast, and lung cancer cells. CXCL17 expression did not impart NIH3T3 cells with oncogenic potential *in vitro*, but CXCL17-expressing NIH3T3 cells could form vasculature-rich tumors in immunodeficient mice. Our data showed that CXCL17-expressing tumor cells increased immature CD11b^+^Gr1^+^ myeloid-derived cells at tumor sites in mice and promoted CD31^+^ tumor angiogenesis. Extensive chemotactic assays proved that CXCL17-responding cells were CD11b^+^Gr1^high^F4/80^−^ cells (∼90%) with a neutrophil-like morphology *in vitro*. Although CXCL17 expression could not increase the number of CD11b^+^Gr1^+^ cells in tumor-burdened SCID mice or promote metastases of low metastatic colon cancer cells, the existence of CXCL17-responding myeloid-derived cells caused a striking enhancement of xenograft tumor formation.

**Conclusions/Significance:**

These results suggest that aberrant expression of CXCL17 in tumor cells recruits immature myeloid-derived cells and promotes tumor progression through angiogenesis.

## Introduction

The tumor microenvironment is the result of an extremely complex series of biological events that depend on tumor cell interaction with the responding host cells [1]. Although the exact components of the tumor microenvironment are heterogeneous in different tumor types and differ according to the stages of tumor progression, the process known as angiogenesis, or the generation of new blood vessels from pre-existing vasculature, is a prominent feature of successful tumor growth in the majority of solid tumors [2], [3]. In fact, a variety of factors produced by both tumor cells and host responding cells have been discovered that regulate angiogenesis [1]–[4], and data emerging from current studies demonstrate that tumor-associated macrophages (TAMs) [1], [4], mesenchymal stem cells (MSCs) [5], and myeloid-derived suppressor cells (MDSCs) [4]–[6] are accumulated at tumor sites and play a pivotal role in tumor angiogenesis, as well as tumor progression and metastasis.

Chemokines are involved in the growth and progression of many tumor types, although they were previously regarded mainly as indispensable chemotactic cytokines for immunity and inflammation [1], [7]. For example, the directional migration of A2056 human melanoma cells was shown by exposure of the C-X-C-type chemokine ligand (CXCL) 8 [8], and both CXCL8 and CXCL5 expression in non-small cell lung carcinoma (NSCLC) has been correlated with tumor angiogenesis [9]. Human CXCL17 (also referred to as dendritic and monocyte chemokine-like protein [DMC] or VEGF correlated chemokine 1 [VCC-1]) has been identified as the latest member of the C-X-C chemokine family following fine structure-based protein analysis and cDNA microarray analysis [10], [11]. Although it was demonstrated that the new C-X-C chemokine induces migration of CD14^+^ monocytes and CD11c^+^ immature dendritic cells (DCs) from human peripheral blood [10] and contributes to potential angiogenesis [11], the role of the chemokine in tumorigenesis remains unclear. Herein, we demonstrate that CXCL17 recruits CD11b^+^Gr1^+^ myeloid-derived cells at tumor sites and promotes angiogenesis and tumorigenesis. Moreover, some human tumor cells expressed CXCL17, and a xenograft tumor model using SCID mice showed rapid tumor formation with rich microvasculature. Therefore, we propose that the aberrant expression of CXCL17 in tumor cells may play a pivotal role in tumor progression.

## Results

### CXCL17 Promotes Tumor Formation without Oncogenic Transformation

**Figure 1:**
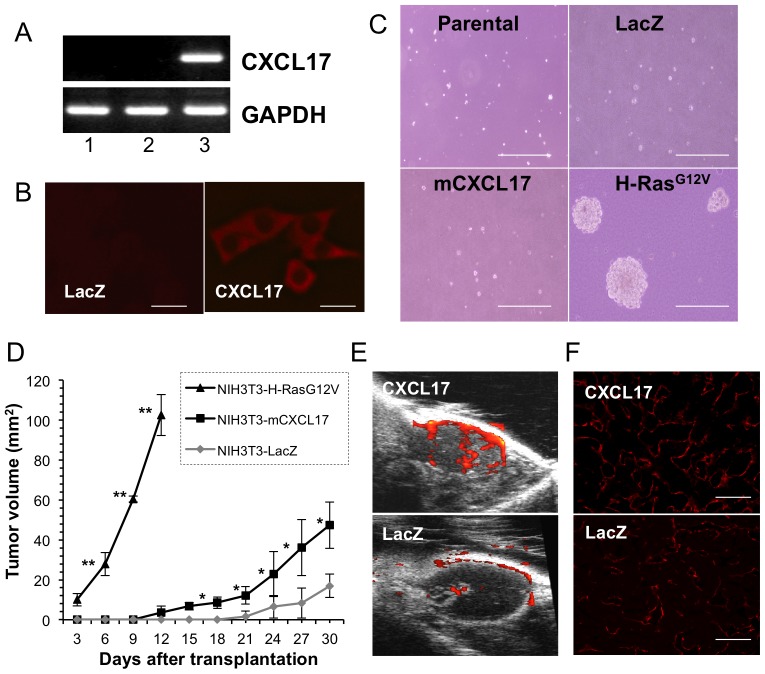
Tumorigenic potential of mouse CXCL17-expressing NIH3T3 cells. (A) Analysis of transduced mCXCL17 mRNA expression in NIH3T3 cells using RT-PCR. Upper panel, CXCL17; lower panel, GAPDH as an internal control. Lane 1, parental cells; lane 2, LacZ-transfected cells; lane 3, mCXCL17-transfected cells. (B) Immunostaining of CXCL17-expressing NIH3T3 cells probed with anti-mCXCL17 Ab. Left, LacZ-transfected cells; right, mCXCL17-transfected cells. Scale bar: 20 um. (C) Anchorage-independent growth assay. Representative phase-contrast microscopic images are shown. Scale bar: 100 um. (D) Transfected NIH3T3 cells (1×10^6^) were transplanted into the subcutaneous space of CHO nude mice and tumor size was measured at the indicated time points. *, P = 0.0154 (mCXCL17 versus LacZ); **, P<0.0001 (mCXCL17 versus H-Ras^V12^); One of two independent experiments with similar results (n = 3−4 per group). (E) Representative Doppler-based ultrasound real-time imaging of differential tumor blood flow. Upper, CXCL17 transfection; lower, LacZ transfection. (F) Immunostaining of tumor from NIH3T3 cells probed with anti-CD31 Ab. Upper, CXCL17-transfected cells; lower, LacZ-transfected cells. Scale bar: 100 um.

A previously published study revealed that human CXCL17-expressing NIH3T3 (hCXCL17-3T3) cells formed tumors in athymic nude mice [11]. This led to the hypothesis that CXCL17 overexpression may provide oncogenic potential to NIH3T3 cells. In order to investigate this hypothesis, we generated mouse CXCL17-3T3 cells by retroviral transduction. RT-PCR analysis showed a CXCL17-specific PCR product (384 bp) in a transfection-dependent manner (Figure 1A). Immunostaining probed by anti-mCXCL17 Ab also showed the protein expression in the cytoplasm of mCXCL17-3T3 cells (Figure 1B). In an effort to determine whether mCXCL17-3T3 cells had acquired the anchorage-independent growth property, a colony formation assay in soft agar was performed [12]. As shown in Figure 1C, no colony was generated in mouse CXCL17-3T3 cells, whereas H-Ras^G12V^-transformants gave rise to numerous and large colonies. The level of proliferation of mCXCL17-3T3 cells was not altered relative to that of LacZ-expressing NIH3T3 (LacZ-3T3) cells under regular adherent culture conditions (data not shown). However, subcutaneous injection of mCXCL17-3T3 cells into nude mice showed more rapid tumor formation than that produced by LacZ-3T3 cells (Figure 1D). The high-power view of the allograft of H-Ras^G12V^-transformed tumor cells showed plump nuclei with abundant mitotic figures, whereas the histology of mCXCL17-3T3 and LacZ-3T3-transfected cells was similar, characterized by a spindle-shaped morphology and less proliferative activity (Figure S1). Moreover, blood flow monitoring by Doppler-based ultrasound real-time qualitative imaging demonstrated abundant signals (orange) in mCXCL17-3T3-derived tumor at an equivalent volume (75 mm^3^, 25% in mCXCL17-3T3 versus 15% in LacZ-3T3; Figure 1E). Immunostaining of mCXCL17-3T3-derived tumor using anti-CD31 Ab also revealed increased formation of vasculature (Figure 1F). These results indicate that CXCL17 does not have oncogenic transformation activity with NIH3T3 cells *in vitro,* but suggest that CXCL17 introduces tumorigenicity through angiogenesis *in vivo.*

### Implication of CXCL17 Expression with Human Cancer

**Figure 2:**
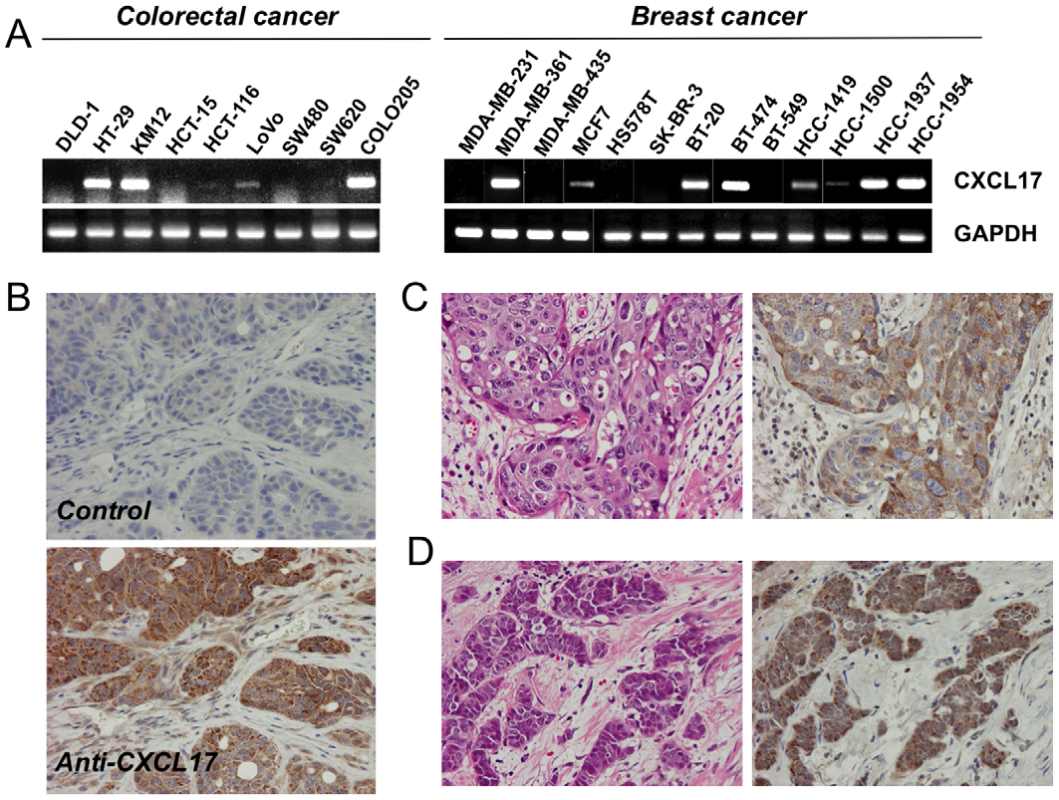
CXCL17 mRNA expression in various human cell lines. (A) Analysis of human CXCL17 mRNA expression in human colon and breast cancer cell lines using RT-PCR. Upper panel, CXCL17; lower panel, GAPDH as an internal control. Cell line names are indicated on each panel. (B) Immunohistochemistry for CXCL17 in the representative xenograft of HT-29 cells, using normal rabbit IgG (upper panel, control) and anti-human CXCL17 IgG (lower panel). Magnification is × 100. The brown color (3,3′-diaminobenzidine) indicates positive staining. Surgically resected colon (C) and breast (D) cancers were probed with anti-human CXCL17 antibodies. Representative images are shown (see *Materials and Methods*). Magnification is × 100. Upper panel, H&E staining; lower panel, anti-human CXCL17 IgG.

In order to determine whether human cancer cells express CXCL17, an RT-PCR assay was performed using 54 human cancer cell lines (colorectal, gastric, renal, breast, non-small cell lung, pancreatic carcinoma, and melanoma) (Figure 2A and 2B, and Figure S2 and S3A). CXCL17 was expressed most frequently in colon and breast carcinoma cell lines, some gastric cell lines, and non-small cell lung cancer and pancreatic carcinoma cell lines, but not in melanoma cell lines. The data from the 54-cell line panel was consistent with the available data measuring CXCL17 expression across nearly 1,000 human cancer cell lines examined by the Cancer Cell Line Encyclopedia project (http://www.broadinstitute.org/ccle/home). Moreover, preliminary immunohistochemical analyses showed that CXCL17 was positive in some clinical specimens (50% in colorectal cancer, 60% in breast cancer, and 30% in non-small cell lung carcinoma) (Figure 2C and 2D, and Figure S3B). Thus, further follow-up data using immunohistochemistry would be helpful in evaluating the protein levels of CXCL17.

**Figure 3:**
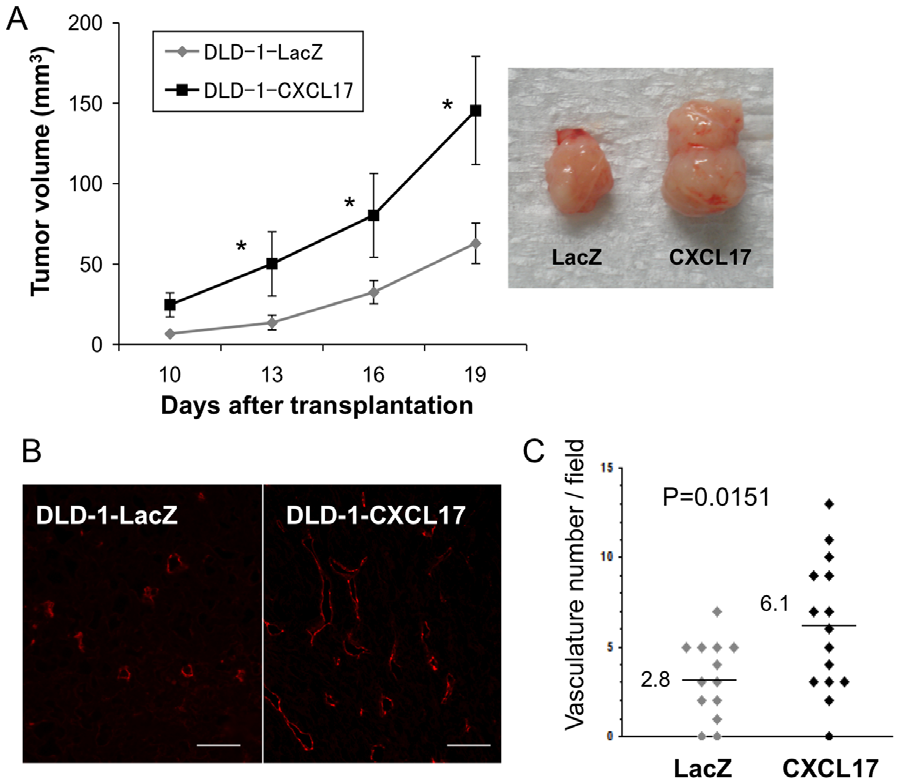
Enhanced tumor formation in CXCL17-expressing DLD-1 colorectal cancer cells. (A) Transfected DLD-1 cells (1×10^6^) were transplanted into the subcutaneous space of C.B-17 SCID mice and tumor volume was measured at the indicated time points (left panel). *, P<0.05 (mCXCL17 versus LacZ transduction: n = 3−4 per group). One of three independent experiments with similar results is shown. Right panel, representative tumors isolated from mice at the end of the observation period. (B) Immunostaining of tumors from DLD-1 cells probed by anti-CD31 Ab. Left, LacZ-transduction; right, CXCL17-transduction. Scale bar: 100 um. (C) The number of CD31-positive vasculatures per field. CD31-positive vessels were counted in five random sections. One of three independent experiments with similar results is shown.

We further investigated whether CXCL17 expression could induce rapid tumor growth of CXCL17-negative DLD-1 colon cancer cells in SCID mice. Mouse CXCL17-expressing DLD-1 (CXCL17-DLD-1) cells were generated and injected into the subcutaneous space of SCID mice (Figure 3A). Strikingly, CXCL17-DLD-1 cells formed tumors more rapidly than control LacZ-DLD-1 cells. There was no difference in cell proliferation among CXCL17-DLD-1, lacZ-DLD-1, and parental cells (data not shown). Similar results were obtained from CXCL17-SW620 cells (Figure S4A). Moreover, the CD31-positive microvasculature increased in CXCL17-DLD-1 tumors (2-fold in number) compared with LacZ-DLD-1 cells (Figure 3B and 3C). Doppler-based ultrasound imaging analysis showed increased blood flow in an early tumorigenic stage in CXCL17-SW620 cells (around 10–15 days; Figure S4B). Stronger blood flow signals were observed in CXCL17-expressing tumors at the equivalent volume (75 mm^3^) (Figure S4C). These results suggest that overexpression of CXCL17 promotes tumor formation *in vivo* through an increase in tumor vessels.

**Figure 4:**
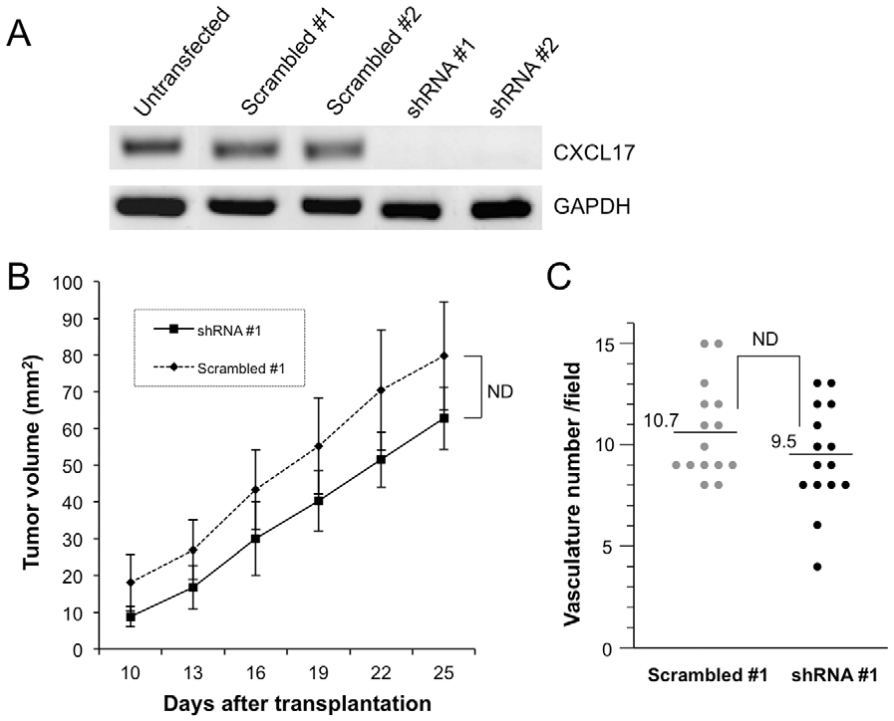
Tumorigenic effect of CXCL17 knockdown in HT-29 cells. (A) Two candidate sequences for shRNA for knocking down CXCL17 expression (shRNA #1 and #2) and corresponding scrambled sequences were also designed (Scrambled #1 and #2). Stable cell lines were isolated and knockdown levels of CXCL17 mRNA were analyzed using RT-PCR. Upper panel, CXCL17; lower panel, GAPDH as an internal control. (B) CXCL17-knockdown HT-29 cells (1 × 10^6^) were transplanted into the subcutaneous space of SHO mice and tumor size was measured at the indicated time points. There was no significant difference between shRNA #1 and Scrambled #1. ND, not different; P = 0.08 (n = 4), One of two independent experiments with similar results is shown. (C) CD31-positive vessels of tumors in xenografts of CXCL17-knockdown HT-29 cells were counted in five random sections. ND, not different (P = 0.19). One of two independent experiments with similar results is shown.

Whether CXCL17 expression affects the metastatic potential of tumor cells is of immense interest in tumor biology. While representative CXCL17-positive colon cancer cells such as HT-29, KM12, and Colon-205 cells showed hematogenous distant metastases with respect to their own properties (see Materials and Methods S1 and Figure S5A), CXCL17-negative DLD-1, SW620, and HCT-15 cells did not show any distant metastases (Figure S5A and S5B). We then addressed whether CXCL17-expressing DLD-1 cells could promote distant organ metastases. The cells were injected into the portal vein of SCID mice (Figure S5C), but metastatic tumor formation was not observed in the liver of mice. Similar results were obtained using CXCL17-expressing SW620 cells (data not shown). Furthermore, in an effort to clarify the role of CXCL17 in regard to tumorigenic potential, we also performed CXCL17-knockdown experiments using HT-29 cells (Figure 4). Our findings showed that CXCL17-knockdown could not significantly impair the aggressive tumorigenic phenotype and angiogenesis. Thus, these results suggest that exogenous CXCL17 expression is unable to alter the less metastatic phenotype of tumor cells to the aggressive phenotype.

### CXCL17 Recruited Immature Myeloid-derived Cells at Tumor Sites

**Figure 5:**
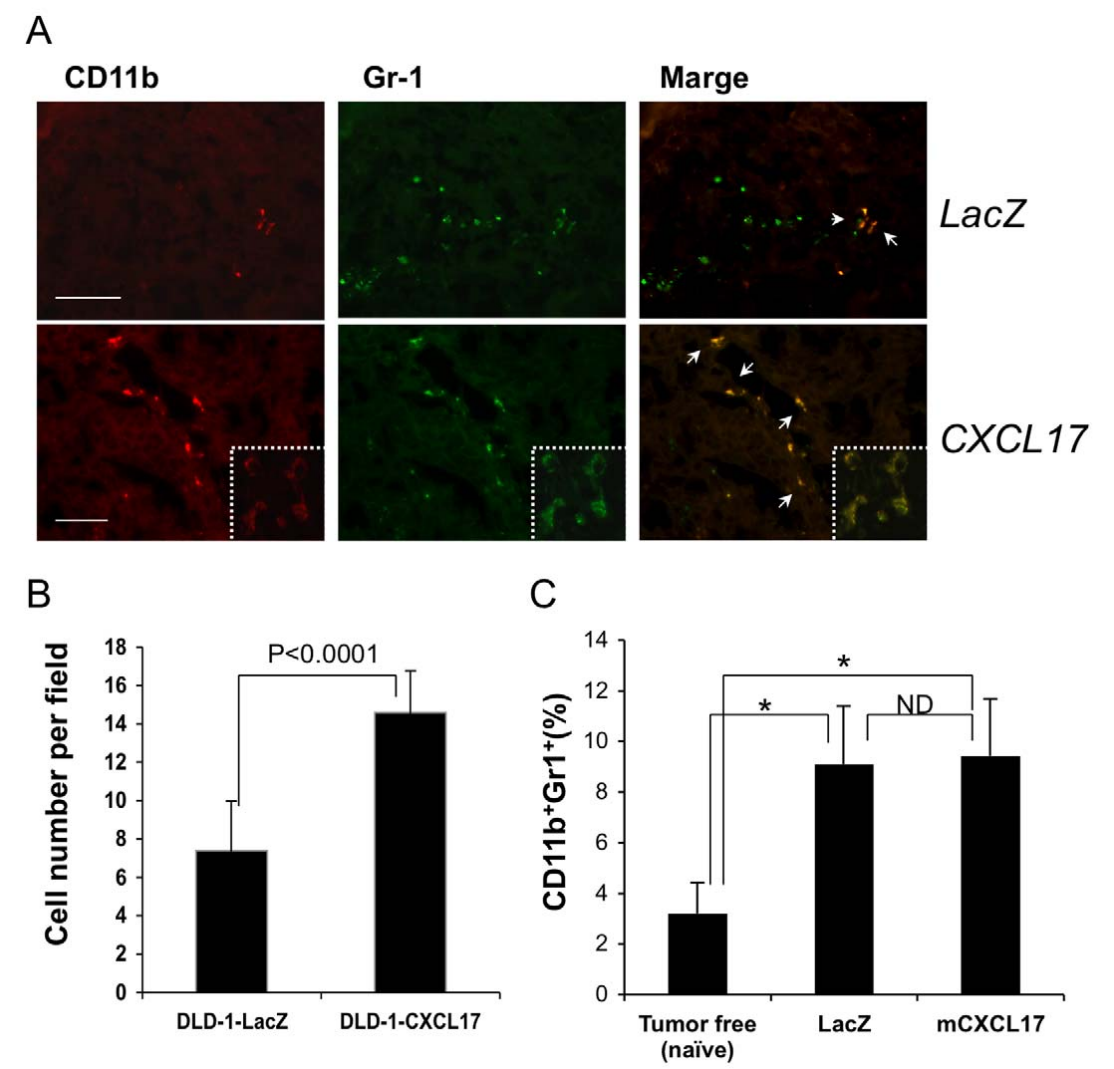
Accumulation of immature myeloid-derived cells in CXCL17-expressing tumor. (A) Tumor sections were stained with anti-CD11b (red) and anti-Gr-1 (green) mAbs. Representative stained images are shown. Upper panels, LacZ transduction; lower panels, CXCL17 transduction. Arrows indicate CD11b^+^Gr-1^+^ cells. Higher magnification images are shown in the lower-right corner (white dotted area). Scale bar: 100 um. (B) The number of CD11b^+^Gr-1^+^ cells per field. CD11b^+^Gr-1^+^ cells were counted in five random sections. One of three independent experiments with similar results is shown. (C) The number of CD11b^+^Gr-1^+^ cells was determined by FACS in the spleen of Colon26 tumor-bearing BALB/c mice. *, P<0.05 (tumor-bearing versus tumor-free naïve mice); ND, not different.

In an effort to examine tumor angiogenesis through CXCL17, we observed cell infiltrates at the edge of CXCL17-DLD-1-transplanted tumors. Those cells were greatly stained by CD11b and Gr-1 mAb (Figure 5A), and some were CD11b and Gr-1 double-positive. In comparison with the LacZ-DLD-1 tumor, CD11b^+^Gr-1^+^ cells had increased in number at tumor sites (2-fold, Figure 5B).

Since it is well known that tumor-bearing conditions significantly increased the population number of CD11b^+^Gr-1^+^ myeloid-derived cells in the spleen of immune-competent mice [6], we further examined whether CXCL17 expression could increase the population number of CD11b^+^Gr-1^+^ myeloid-derived cells. CXCL17- or LacZ-expressing murine Colon26 cancer cells [13] were transplanted into the subcutaneous space of syngeneic BALB/c mice. CXCL17- and LacZ-Colon26 cells formed tumors, although there was no difference in tumor size (data not shown). In comparison with naïve tumor-free mice, the population of CD11b^+^Gr-1^+^ cells could increase in the tumor-bearing mice, although the populations of CXCL17- and LacZ-Colon26 tumors were not segregated (Figure 5C). Thus, these results suggest that CXCL17 expression in tumor cells may recruit CD11b^+^Gr-1^+^ myeloid-derived cells at tumor sites, but not increase the population size of CD11b^+^Gr-1^+^ cells.

**Figure 6:**
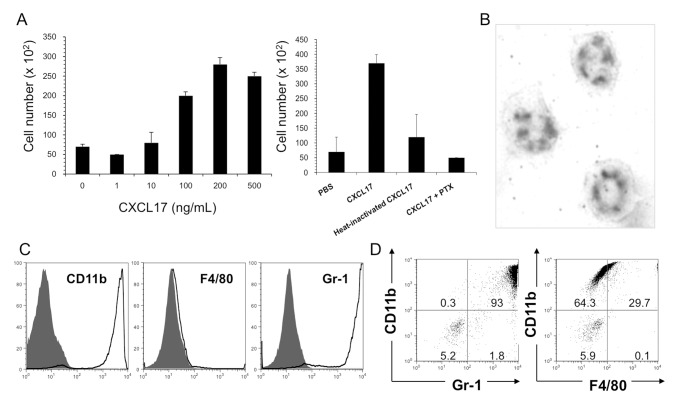
Characterization of CXCL17-responding cells *in vitro*. (A) Spleen cells were isolated from SCID mice and used for the chemotaxis assay (see *Materials and Methods*). Left panel, dose-dependent responses with recombinant CXCL17; right panel, chemotactic responses following various treatments. (B) Morphology of CXCL17-responding cells. CXCL17-responding cells were adhered onto a glass slide by centrifugation and then subjected to Wright-Giemsa staining. (C and D) Flow cytometric analysis of mouse CXCL17-responding cells (in the lower chamber of the chemotaxis assay). mCXCL17-responding cells were stained with PE-conjugated anti-mouse CD11b, FITC-conjugated anti-mouse F4/80 and FITC-conjugated anti-mouse Gr-1 mAbs, and then analyzed using flow cytometry. Shaded histograms represent staining with isotype-matched mAbs.

We then set out to identify the type of murine cells that could respond preferentially to CXCL17 *in vitro*. Spleen cells were isolated from naïve SCID mice since CD11b^+^Gr-1^+^ myeloid-derived cells were greatly increased in the mice (Figure S6A). A chemotaxis assay was then performed using recombinant CXCL17. As shown in Figure 6A (left panel), some spleen cells definitely responded to recombinant mCXCL17 in a dose-dependent manner. Cell migration was blocked by heat-inactivation of the recombinant protein and treatment with pertussis toxin (PTX) (Figure 6A, right panel). The majority of CXCL17-responding cells showed a neutrophil-like morphology (Figure 6B). Similar increases in CXCL17-responding cells were also observed in tumor-bearing BALB/c mice in which CD11b^+^Gr-1^+^ accumulated greatly by the transplantation of unmanipulated Colon26 cells (Figure S6B).

Furthermore, in an effort to identify CXCL17-responding cells, FACS analysis was performed using migrated cells by recombinant mCXCL17. Responding cells were predominantly CD11b-positive and Gr-1-positive (93%), but F4/80-negative (Figure 6C and 6D). Similar results were obtained with recombinant human CXCL17 (Figure S7A) and F4/80-positive cells that remained in the upper chamber of the chemotaxis assay (Figure S7B). We further examined whether these cells might be segregated with monocytic myeloid cells in the spleen of mice. Recombinant mouse CCL2 (MCP1) was used as a representative CCR2 ligand, which plays a pivotal role in the recruitment of monocytes at tumor sites [14]–[16]. CCL2-responding cells showed CD11b^high^Gr-1^+^ monocyte-like phenotypes, compared with CXCL17-responding cells (Figure S7C). Thus, these results suggest that CD11b^+^Gr-1^high^F4/80^−^ myeloid-derived cells (neutrophil-like) are sensitive to CXCL17.

### CXCL17-responding Myeloid-derived Cells Promote Tumor Formation and Angiogenesis

**Figure 7:**
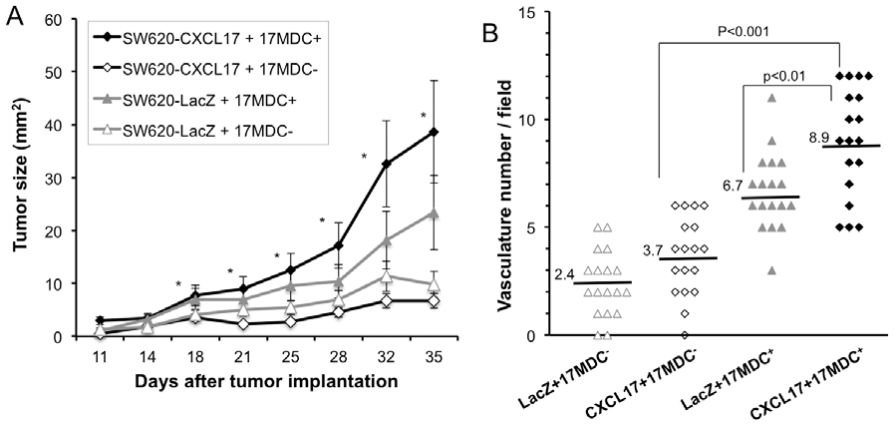
CXCL17-responding myeloid cells promote tumor formation and angiogenesis. (A) CXCL17-responding myeloid-derived cells (17MDC^+^, 1×10^5^) were collected from the lower chamber of the chemotaxis assay (4-hr incubation), and inoculated subcutaneously into SCID (SHO) mice together with SW620-CXCL17 or -LacZ cells (1×10^6^). CXCL17-unresponding myeloid-derived cells (17MDC^−^, 1×10^5^) were used as control cells. Tumor growth was monitored every 3–4 days following tumor implantation. *, P<0.05 (Kruskal-Wallis test, SW620-CXCL17+17MDC^+^ versus SW620-CXCL17+17MDC^−^, SW620-LacZ +17MDC^−^ at day 18–35 following tumor implantation). One of two experiments with similar results is shown. (B) CD31-positive vessels of tumors from SW620-CXCL17 cells with CXCL17-responding cells were counted in five random sections. One of two independent experiments with similar results is shown.

We further addressed whether CXCL17-responding myeloid-derived cells could promote tumorigenesis of CXCL17-expressing tumor cells. Migrated myeloid-derived cells in the CXCL17-dependent manner were collected from the lower chamber of the chemotaxis assay (4-hr exposure), and these cells together with CXCL17-expressing SW620 cells were injected subcutaneously into SCID mice (Figure 7A). In comparison with the use of CXCL17-unresponding cells (from the upper chamber of the chemotaxis assay), CXCL17-recruited cells caused a striking enhancement in tumor formation of CXCL17-SW620 cells. Interestingly, CXCL17-responding cells could also moderately promote tumor formation of the control LacZ-SW620 cells. Moreover, CD31^+^ vasculatures in the CXCL17-SW620 tumor also increased in number in the presence of CXCL17-responding myeloid-derived cells (Figure 7B). Thus, these results demonstrate that CXCL17-responding myeloid-derived cells promote tumor growth in conjunction with angiogenesis. Regarding CXCL17-mediated angiogenesis, CXCL17 increased the levels of VEGF-A in vascular endothelial cells [11]. While VEGF-A expression and cell migration was observed in vascular endothelial cells (Figure S8), VEGF-A was not induced in the immediately migrated myeloid-derived cells by CXCL17 (data not shown). These results suggest that CXCL17-recruited myeloid-derived cells may need to be modified for angiogenic potential in the tumor microenvironment.

## Discussion

Herein, we have demonstrated the potential role of chemokine CXCL17 in tumor formation. The remarkable features presented in this study include: 1) CXCL17-producing cells increased angiogenesis *in vivo* and formed tumors, 2) some of the human cancer cells expressed CXCL17, 3) they recruited immature CD11b^+^Gr-1^+^F4/80^−^ myeloid-derived cells, and 4) the recruited myeloid-derived cells by CXCL17 could promote tumor growth *in vivo*.

While CXCL17 was identified as a potential chemokine to human immature DCs and monocytes [10], it has been shown to act as a tumor-forming factor [11]. In the latter case, since it is well known that NIH3T3 cells are vulnerable to cell transformation [12], [17], [18], our first question focused on whether CXCL17 plays a role as a potential oncogene or whether the unidentified receptor on NIH3T3 cells might be activated in an autocrine manner. Constitutive stimulation from G-protein coupled receptors (GPCRs) (e.g., KSHV vGPCR) could link cell transformation and proliferation, and tumorigenicity [19], [20]. To address the above question, a colony formation assay was performed in soft agar, and transformed or constitutively activated 3T3 cells should have formed colonies in an anchorage-independent fashion. However, CXCL17-expressing 3T3 cells did not form any colonies (Figure 1C) or transformation foci (data not shown), and those cells could only form tumors in nude mice (Figure 1D). These results demonstrate that CXCL17 has less oncogenic activity, and perhaps suggest that NIH3T3 cells do not express the CXCL17 receptor.

We could successfully monitor tumor angiogenesis using Doppler-based high-resolution ultrasonography (Figure 1E) without killing the experimental mice. The analyzed imaging data correlated well with the CD31-positivity in specimens of CXCL17-expressing tumors (Figure 1F), suggesting that CXCL17-mediated tumorigenicity is based on the host-tumor interaction and angiogenesis. To extend the implication of CXCL17 expression to human cancer biology, CXCL17 expression was screened in various cancer cell lines (approximately 50 cell lines, Figure 2 and Figure S2). Previous reports showed that CXCL17 was expressed in normal stomach, trachea, and lungs (bronchiolar epithelium and a subset of alveolar lining cells) [10], although some colon and breast tumors were CXCL17-positive [11]. In our data, CXCL17 was expressed frequently in colorectal, breast, and lung (non-small cell type) cancer cell lines and some specimens from those patients. Additionally, our study showed that CXCL17 was also expressed in some gastric and pancreatic carcinoma cell lines, but not in melanoma cell lines. Although CXCL17-positive cancer cell lines had aggressive phenotypes in xenogeneic transplantation studies using SCID mice, even overexpression of CXCL17 in less metastatic cell lines without CXCL17 expression led to metastatic growth in the liver. We also performed CXCL17-knockdown experiments using HT-29 cells. However, CXCL17-knockdown had a lower effect on tumorigenicity and angiogenesis (Figure 4). These findings suggest that oncogenic transforming events play a critical role in the phenotypic differences. Therefore, it is possible that CXCL17 expression may be acquired in the late process of cancer progression (or development). The stimuli necessary to effect CXCL17 expression in some cancer cells and the manner in which these stimuli are involved in cancer progression remain to be elucidated.

Various types of myeloid cells have been demonstrated to promote tumor progression by direct immune suppression [21] and the production of angiogenic factors and growth factors [21]–[23]. With regard to tumor formation, we found accumulations of immature myeloid cells in the tumor edge of CXCL17-positive colon cancer cell lines, but not CXCL17-negative cell lines. Overexpression of CXCL17 clearly increased the number of CD11b^+^Gr-1^+^ myeloid-derived cells at tumor sites and was coupled with enhanced tumorigenicity and increased vasculature *in vivo* (Figure 3). It may be worth considering the relationship between CXCL17-responding myeloid-derived cells and myeloid-derived suppressor cells (MDSCs). MDSCs still represent a heterogeneous population of immune suppressive cells in humans and mice and are excessively produced in cancer, which can be either monocytic (Ly-6C^+^) or granulocytic (Ly-6G^+^), and function as systemic immune suppressors and promoters of tumor angiogenesis [4], [6]. In humans, a specific marker that will unequivocally identify MDSC has yet to be identified, and this population has not been defined in a uniform manner [24]. Therefore, extensive studies are needed to determine the relationship between mouse and human MDSCs. Recently, Hiraoka *et al.* [25] reported that myeloid-derived cells are accumulated in the intraepithelial tissue of early human pancreatic carcinogenesis, and that CXCL17 may be involved in an anti-tumor immune response. CXCL17-expressing Colon26 cells could form tumors rapidly *in vivo*, and tumor growth between CXCL17- and LacZ-expressing Colon26 cells was not segregated. This dichotomy may have resulted from the specific properties derived from the different cell lines. Additionally, it has previously been shown that MDSCs can enter tumors and differentiate into mature macrophages (TAMs) or neutrophils [26], and that neutrophils placed in intratumoral conditions are imparted with tumoricidal or tumorigenic potential (by the presence or absence of local TGF-beta) [27]. Thus, it is also likely that the nature of recruited myeloid-derived cells and immune cells may be altered by the difference in tumor-microenvironment between immunocompetent and immunodeficient conditions.

Our results and those of other researchers indicate that CXCL17 may have a dual biological role in tumorigenesis. While the migration of immature myeloid cells was induced at lower concentration levels (0.1–0.2 ug/mL), that of endothelial cells needed a high concentration of CXCL17 (1.0–2.5 ug/mL). The latter case resulted from the physiological conditions for a chemokine. Similar endothelial cell activation through CXCL17 was demonstrated by high-efficient adenoviral transduction of CXCL17 cDNA, in which endothelial cells demonstrated increased levels of VEGF-A, basic FGF, and PDGF [11]. These growth factors have been shown to promote the proliferation and migration of endothelial cells [28]–[30]. This case might result from those factors induced by CXCL17. Nonetheless, these findings suggest that two types of CXCL17 receptor with high and low affinity may exist, or that levels of the specific receptor might be high or low among responding cells. At the very least, it seems likely that local CXCL17 can activate preexisting endothelial cells after differentiation from recruited endothelial progenitors. To ascertain this dual aspect in tumor microenvironments, identification and characterization of the CXCL17 receptor is needed.

Chemokines are emerging as key mediators in the recruitment of a number of different cell types to the tumor microenvironment [7], [15], [31], [32]. These infiltrating cells provide a secondary source of chemokines that could affect tumor growth and angiogenesis [7], [15], [31], [32]. Recent studies have revealed that myeloid-derived cells should play an important role in angiogenesis by producing various angiogenic factors [4], [6], [22], [23]. Furthermore, accessory cells such as neutrophils could promote angiogenesis and alter immune responses in tumor microenvironments [27], [31]. Our studies support the view that the aberrant expression of CXCL17 in human cancer cells recruits immature myeloid-derived cells in mice and promotes tumor progression through angiogenesis. Although the mechanism by which the *CXCL17* gene is activated in malignant cells remains unknown, CXCL17 seems to be involved in the complex tumor microenvironment in some human cancers. Further understanding of the properties of CXCL17 expression and homing cells should provide important clues in efforts to elucidate the components of the tumor microenvironment.

## Materials and Methods

### Ethics Statements

All animal experiments in this study were approved by the Animal Ethics Review Board of Jichi Medical University (#10–132) and Takasaki University of Health and Welfare (#1119), performed in accordance with the institutional guide for laboratory animals, and followed the principles of laboratory animal care formulated by the National Society for Medical Research. All clinical samples were procured and used according to a protocol approved by the Medical Ethics Committee of Gunma University (followed by principles detailed in the Declaration of Helsinki) from the Pathology Archive of Gunma University and its affiliated hospital. All patients provided written informed consent for the use of their tissues, and all patient information associated with this study was obtained in de-identified format.

### Animals, Cells and Reagents

BALB/c, BALB/c AJcl-nu/nu (BALB/c nude) and C.B-17/Icr-scid/scidJcl (C.B-17 SCID) mice (8–12 weeks old) were purchased from CLEA Japan, Inc. (Tokyo, Japan), and NOD C.B-17-Prkdc^scid^/J (NOD/SCID) and Crlj:SHO-Prkdc^scid^Hr^hr^ (CHO) mice (8–12 weeks old) were purchased from Charles River Japan (Yokohama, Japan).

NIH3T3 cells and all human cancer cell lines were obtained from the American Type Culture Collection (Rockville, MD) and the Health Science Research Resources Bank (HSRRB) (Osaka, Japan). Cells were maintained in Dulbecco’s modified Eagle’s medium (DMEM, Sigma-Aldrich, St. Louis, MO), RPMI1640 medium (Sigma-Aldrich), McCoy’s 5A medium (Invitrogen, GIBCO, Gaithersburg, MD), or Leibovitz L-15 medium (Sigma-Aldrich), supplemented with 10% heat-inactivated fetal calf serum (FCS) and additional components appropriate for each cell line [33], [34]. The cultures were maintained in a humidified atmosphere containing 5% CO_2_ and 95% air at 37°C.

Anti-mouse CXCL17 (monoclonal, clone 510614) antibody was obtained from R&D Systems (Minneapolis, MN). Anti-human CXCL17 (polyclonal rabbit IgG, VCC-1 [H-43]: sc-292019) and anti-CD31 antibodies were purchased from Santa Cruz Biotechnology (Santa Cruz, CA). Flow cytometric analysis involved the use of phycoerythrin (PE)-conjugated anti-mouse CD11b mAb, fluorescein isothiocyanate (FITC)-conjugated anti-Gr-1 (Ly-6G) mAb, FITC-conjugated anti-mouse F4/80 mAb, and isotype-matched IgG controls, all of which were purchased from eBioscience (San Diego, CA).

Pertussis toxin (PTX) was obtained from Sigma-Aldrich (St. Louis, MO). Recombinant mouse and human CXCL17 (DMC/VCC1), and recombinant mouse CCL2 (MCP-1) were purchased from R&D Systems and PeproTech (Rocky Hill, MN), respectively.

### RT-PCR and cDNA Cloning

Total RNA was extracted from cells using Isogen® (Nippon Gene, Toyama, Japan). Two micrograms of total RNA were used for first-strand synthesis using SuperScript III™ reverse transcriptase (Invitrogen, Carlsbad, CA). PCR was then performed using ExTaq polymerase (Takara Bio, Ohtsu, Japan). The full-length encoding sequences of mouse CXCL17 (GenBank, NM_153576) and human CXCL17 (GenBank, NM_198477) were amplified from lung-derived cDNA of mice and human MKN-1 cells using the following respective primers: mCXCL17 sense: 5′-ATG AAG CTT CTA GCC TCT CCC-3′; mCXCL17 anti-sense: 5′-CTA TAA GGG CAG CGC AAA GCT TGC-3′; hCXCL17 sense: 5′-ATG AAA GTT CTA ATC TCT TCC CTC-3′; hCXCL17 anti-sense: 5′-CTA CAA AGG CAG AGC AAA GCT TCT TAG C-3′. The PCR product was then inserted into the TOPO® PCR Cloning vector (pCR2.1, Invitrogen, CA). Following confirmation of the nucleotide sequence, the *EcoR*I cDNA fragments were inserted into the pQCXIN retroviral vector (Clontech, Palo Alto, CA) to generate pQCXIN-mCXCL17 and pQCXIN-hCXCL17, respectively. The retroviral expression plasmids for H-Ras^G12V^ (pBabe-H-Ras^G12^; pBabe-puro in control) have been previously described [35].

For expression analysis, the aforementioned and following primers were used: glyceraldehyde-3-phosphate dehydrogenase (GAPDH) sense: 5′-GTA TCG TGG AAG GAC TCA TG-3′; and GAPDH anti-sense: 5′-AGT GGG TGT CGC GCT GTT GAA G-3′; human VEGF-A sense: 5′-ATG ACG AGG GCC TGG AGT GTG-3′; human VEGF-A anti-sense: 5′-CCT ATG TGC TGG CCT TGG TGA G-3′. PCR conditions for each set of primers included initial treatment at 95°C for 2 min, followed by 35 cycles consisting of denaturation at 95°C for 15 sec, annealing at 57°C for 30 sec, and then extension at 72°C for 2 min. PCR products were analyzed using a 1% agarose gel.

### Transfection, Soft Agar Colony Formation Assay, and Cell Proliferation Assay

To generate CXCL17-expressing cells, GP2-293 packaging cells (Clontech) were cotransfected with pQCXIN-mCXCL17 and pVSV-G (Clontech), a plasmid encoding the viral envelope glycoprotein (VSV-G) of the vesicular stomatitis virus, using Lipofectamine 2000 (Invitrogen). Supernatants from transfected GP2-293 were incubated with ∼50% confluent cells in the presence of polybrene (8 ug/ml final concentration; Sigma-Aldrich). Transduced cells were propagated and maintained in medium containing G418 geneticine (Sigma-Aldrich, final 800 ug/ml) or puromycin (Sigma-Aldrich, final 15 ug/ml). Similar retroviral transductions were conducted for hCXCL17, LacZ, and H-Ras^G12V^ cDNA, and the establishment of firefly (*Photinus pyralis*) luciferase-expressing cells has been previously described [13], [34].

For soft agar colony formation assays, cells (1×10^4^) in DMEM containing 10% FCS were suspended in 0.4% agarose (SeaPlaque® Agarose; Takara Bio, Japan). The cell suspension was then layered onto a solidified 0.53% agarose bottom layer containing 10% FCS-DMEM in a 35-mm plate and incubated at 37°C and 5% CO_2_ for 14–21 days until evaluation. Each assay was performed in duplicate and repeated three times with similar results.

For cell proliferation assays, cells (1×10^5^ per well of a 12-well plastic plate in triplicate) were seeded and incubated for the indicated time points, and then adherent cells were counted using a hemocytometer with trypan blue exclusion.

### Knockdown of CXL17

To knock down human CXCL17 mRNA, the following target sequences were selected: shRNA#1, 5′ TAA GAA GCT TTG CTC TGC CTT TGT A-3′; shRNA#2, 5′- GTA GCT TCC TAG CTA GTG T-3′. These were driven from pBAsi-hU6 Pur DNA plasmid (Takara Bio, Ohtsu, Japan). Plasmid DNA was transfected into HT-29 cells and stable CXCL17-knockdown cells were established in medium containing puromycin (Sigma-Aldrich, final 15 ug/ml). Several clones were isolated per target sequence and CXCL17 expression levels were monitored by RT-PCR. Notably, cell proliferation properties did not alter among parental and transfected cells (data not shown).

### Flow Cytometry and Immunofluorescence Staining

For the flow cytometric analysis, cells (1×10^6^) were washed with PBS and incubated with mAb for 30 min at 4°C. Following washing with 0.1% FCS-PBS, cells (5×10^4^) were analyzed using FACSCalibur (Becton Dickinson, Mountain View, CA) and FlowJo analysis software (Tree Star, San Carlos, CA).

For immunofluorescence staining, removed tumor specimens were fixed with 4% paraformaldehyde and embedded in Tissue-Tek® Optimal Cutting Temperature (OCT) compound (Sakura Finetek, Tokyo, Japan). Frozen tissue sections (5 um) were probed with anti-CD31, followed by appropriate Alexa Fluor 594-labelled secondary antibodies (Invitrogen; diluted 1∶500 in 0.1% BSA for 30 min at room temperature). In sections stained with anti-CD31 antibody, CD31-positive tubular structures within the tumor were considered blood vessels. CD31-positive vessels were counted in five random sections.

Immunohistochemical analyses used xenografts of HT-29 (colon cancer), NCI-H460 (non-small cell lung cancer), and A549 (non-small cell lung cancer) cell lines. These were obtained from the American Type Culture Collection (Rockville, MD). Additionally, surgically resected specimens from human lung, breast, and colon cancer (10 cases each, all were adenocarcinoma) were examined. Tissue samples were fixed with 4% formaldehyde for one day and then embedded in paraffin routinely. Three-um-thick sections were prepared and stained with hematoxylin and eosin (H&E). Adjacent sections were reacted with primary antibody to human CXCL17 (2 ug/ml, Santa Cruz Biotechnology) overnight at 4°C. For coloration, a commercially available biotin-streptavidin immunoperoxidase kit (Histofine, Nichirei, Tokyo, Japan) and 3,3′-diaminobenzidine were employed, and counterstained lightly with hematoxylin. Prior to immunostaining, specimens required antigen retrieval by autoclaving (120°C, 10 min) in 10 mM citrate buffer (pH 6.0). Specimens incubated with normal rabbit IgG (2 ug/ml) *in lieu* of the primary antibody, and those not treated with the primary antibody, were utilized as negative controls.

### Tumor Transplantation Model

Cells in exponential growth phase were harvested by trypsinization and washed twice in PBS prior to injection. C.B-17 SCID mice were treated by injection of anti-asialo GM1 Abs (100 mg/body, Wako, Japan) into the peritoneal cavity one day prior to the operation. For the subcutaneous injections, cells (1 × 10^6^) were injected into the subcutaneous space of mice (the hind limb or the abdomen) [13], [36].

### In vivo Imaging

*In vivo* tumor progression at the skin was examined using the Vevo770 high-resolution ultrasound (US) system with contrast mode software (VisualSonics, Toronto, Ontario, Canada). During the US imaging session, the animals were anesthetized with a 2%/98% isoflurane/oxygen mixture, and body temperature was monitored and maintained at 37°C using a warming plate. Coupling gel was applied to the tumor area, and 3-dimensional (3D) mode and power Doppler scout images established a field of view with vascularity present. To enhance vessel imaging, liposomal microbubbles (approximately 0.8 um in diameter; 5×10^7^/ml) [37] were used as ultrasound contrast agents, which comprise small volumes of gas surrounded by a stabilizing lipid shell (1 ug/ul). Each bolus injection of microbubbles (200 ul) was administered into the tail vein of tumor-bearing mice.

### Chemotaxis Assay

Spleen cells isolated from mice were used for chemotaxis assays. Cells (1×10^6^ cells/24 well-plate or 5×10^6^ cells/6 well-plate) were placed on top of the 3-um pore size filters in duplicate, whereas 0.2% BSA-containing RPMI1640 with and without chemokines were placed into the lower chamber (exposed at the indicated concentrations). Following 4 hr at 37°C, migrated cells that had fallen to the bottom of the plate were counted using a hemocytometer and their phenotypes were analyzed by a flow cytometer. PTX treatment occurred at a concentration of 100 ng/ml for 2 hr, and heat-inactivation of recombinant CXCL17 was performed at 95°C for 1 min. Two or three independent experiments were performed with similar results.

### Statistical Analysis

P values based on the two-sided Student’s *t* test, Mann-Whitney U test, or Kruskal-Wallis test were obtained using the Instat 3 software package (GraphPad, San Diego, CA). Differences between groups were considered significant if P<0.05.

## Supporting Information

Figure S1 **Cell morphology of allografts derived from CXCL17-expressing NIH3T3 cells.** Specimens from allografts were stained using H&E (Magnification, x 40; Scale bar, 50 um). Transplanted cells are indicated in each panel.
(TIF)

Figure S2 **CXCL17 mRNA expression in various human cell lines.** (A) Analysis of human CXCL17 mRNA expression in human cancer cell lines using RT-PCR. Upper panel, CXCL17; lower panel, GAPDH as an internal control. Cell line names are indicated on each panel.
(TIF)

Figure S3 **Immunohistochemistry for CXCL17 in the xenograft tumor and in surgically resected specimens from human lung adenocarcinoma.** (A) Immunohistochemistry for CXCL17 in the xenograft tumor of A549 and NCI-H460 cells. (B) Surgically resected lung cancers were probed with anti-human CXCL17 antibodies. Magnification is x 100. The brown color (3,3′-diaminobenzidine) indicates positive staining. Left panels, H&E staining; middle panels, staining with normal rabbit IgG; right panels, anti-human CXCL17 IgG.
(TIF)

Figure S4 **Enhanced tumor formation in CXCL17-expressing SW620 colon cancer cells.** (A) CXCL17-transfected SW620 cells (1×10^6^) were transplanted into the subcutaneous space of C.B-17 SCID mice and tumor volume was measured at the indicated time points by Vevo770. (B) Blood flow analysis using Vevo770 Doppler-based ultrasound imaging analysis in CXCL17-SW620 cells. It is worth noting that tumors of CXCL17-expressing SW620 cells showed increased blood flow signals in the early tumorigenic stage, but not those of control LacZ-SW620 cells. (C) Stronger blood flow signals in CXCL17-SW620 tumors compared to LacZ-SW620 tumors at the equivalent tumor volume (75 mm^3^).
(TIF)

Figure S5 **Metastatic potential in CXCL17-expressing human cancer cell lines.** (A) Luciferase-expressing colon cancer cells were injected into the left ventricle of NOD/SCID mice. Metastatic growth was monitored by tumor-derived photons *in vivo*. Representative images around 30–40 days following tumor injection are shown. It is worth noting that DLD-1, SW620 and HCT-15 cells were less metastasized than HT-29, KM-12 and Colo205 cells. (B) *Ex vivo* luminescent inspection of metastasized organs after intra-cardiac injection of tumor cells. (C) Luciferase and CXCL17 doubly expressing DLD-1 cells were injected into the portal vein of SCID mice and then examined by *in vivo* luminescent imaging.
(TIF)

Figure S6 **Increase in CXCL17-responding cells in tumor-bearing BALB/c mice.** Parental (unmanipulated) Colon26 cells were transplanted subcutaneously into BALB/c mice, and tumor-bearing conditions in BALB/c mice conditions were generated (BALB/c Tumor, at 15–20 days after implantation). (A) Spleen cells were isolated from the indicated mice and the number of CD11b^+^Gr-1^+^ cells was determined by a flow cytometer. It is interesting to note that CD11b^+^Gr-1^+^ cells in BALB/c Tumor increased more than in naïve tumor-free mice. (B) Spleen cells were isolated from the indicated mice and used for the chemotaxis assay using mouse recombinant CXCL17. *, P<0.001 (n = 4). One of two independent experiments with similar results is shown.
(TIF)

Figure S7 **Flow cytometric analysis of CXCL17-responding cells.** (A) Spleen cells were isolated from SCID mice and used for the chemotaxis assay using human recombinant CXCL17. (B) Human recombinant CXCL17-responding cells were stained with PE-conjugated anti-mouse CD11b, FITC-conjugated anti-mouse F4/80 and FITC-conjugated anti-mouse Gr-1 mAbs, and then analyzed using flow cytometry. Isotypes, staining with PE-conjugated and/or FITC-conjugated isotype-matched control IgGs; Upper chamber, remaining cell population; lower chamber, responding cell population. (C) Differential population of CXCL17-responding cells from CCL2-responding cells. The dotted rectangle represents the major population preferentially responding to the indicated chemokine.
(TIF)

Figure S8 **Enhanced migration of HUVECs with CXCL17.** (A) RT-PCR analysis of VEGF-A mRNA expression in HUVECs following recombinant hCXCL17 treatment. HUVECs were exposed to CXCL17 at the indicated concentrations for 10 hr. Upper panel, VEGF-A; lower panel, GAPDH as an internal control. (B) Migration assay of HUVECs in the presence of recombinant hCXCL17. Lines represent reference points for the scratch with a pipette tip. One of two independent experiments with similar results is shown.
(TIF)

Materials and Methods S1 (DOC)
