# Supplementary figures and images for: CXCL17 Expression by Tumor Cells Recruits CD11b+Gr1highF4/80− Cells and Promotes Tumor Progression
Source: PLoS One. 2012 Aug 29;7(8):e44080. doi: 10.1371/journal.pone.0044080 (PMC3430639; doi:10.1371/journal.pone.0044080)

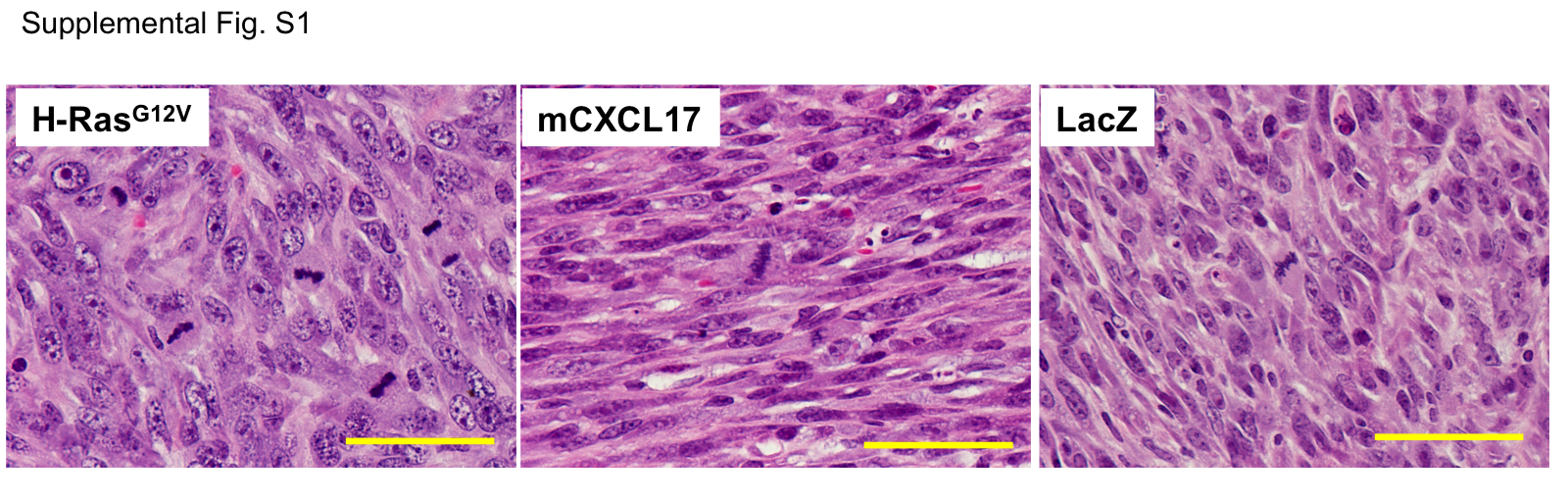

Supplement: Figure S1 — Cell morphology of allografts derived from CXCL17-expressing NIH3T3 cells. Specimens from allografts were stained using H&E (Magnification, x 40; Scale bar, 50 um). Transplanted cells are indicated in each panel. (TIF) [file pone.0044080.s001.tif]

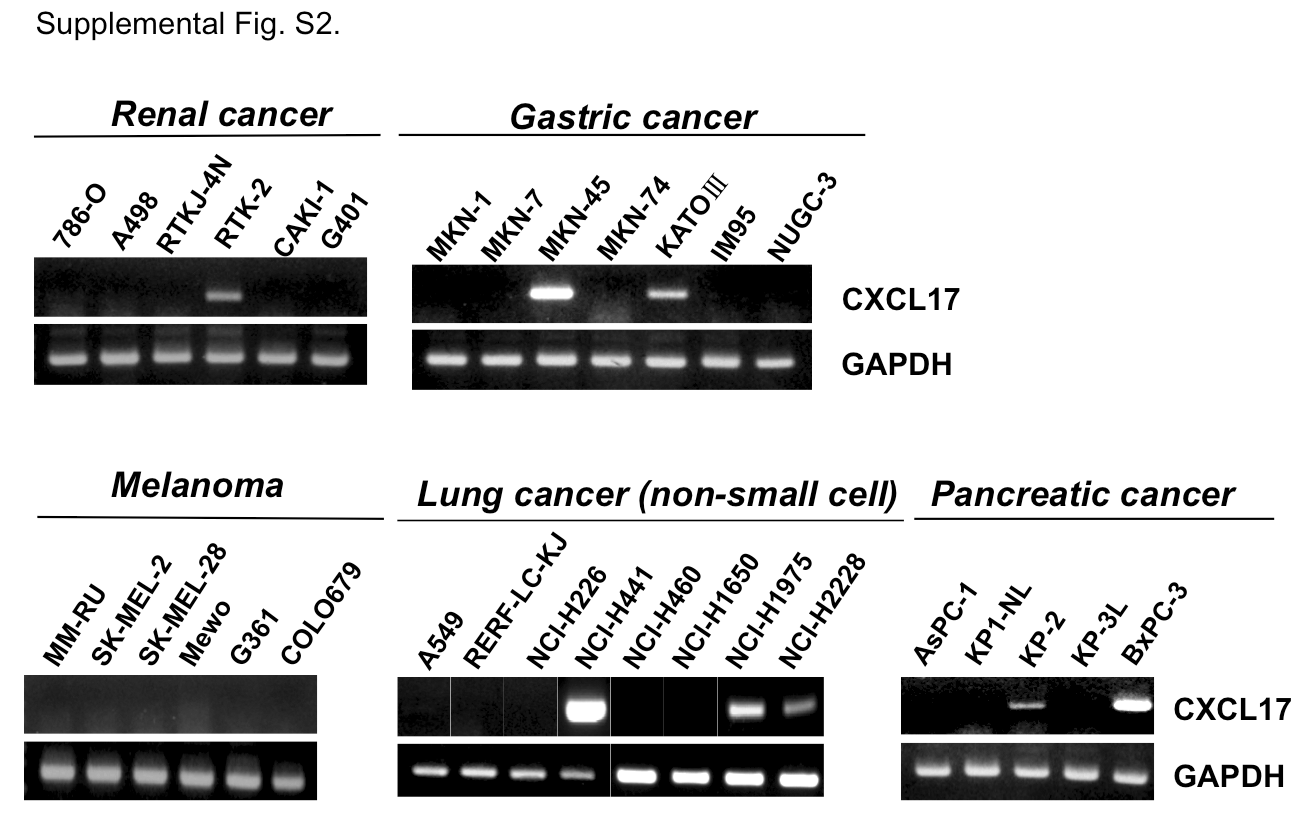

Supplement: Figure S2 — CXCL17 mRNA expression in various human cell lines. (A) Analysis of human CXCL17 mRNA expression in human cancer cell lines using RT-PCR. Upper panel, CXCL17; lower panel, GAPDH as an internal control. Cell line names are indicated on each panel. (TIF) [file pone.0044080.s002.tif]

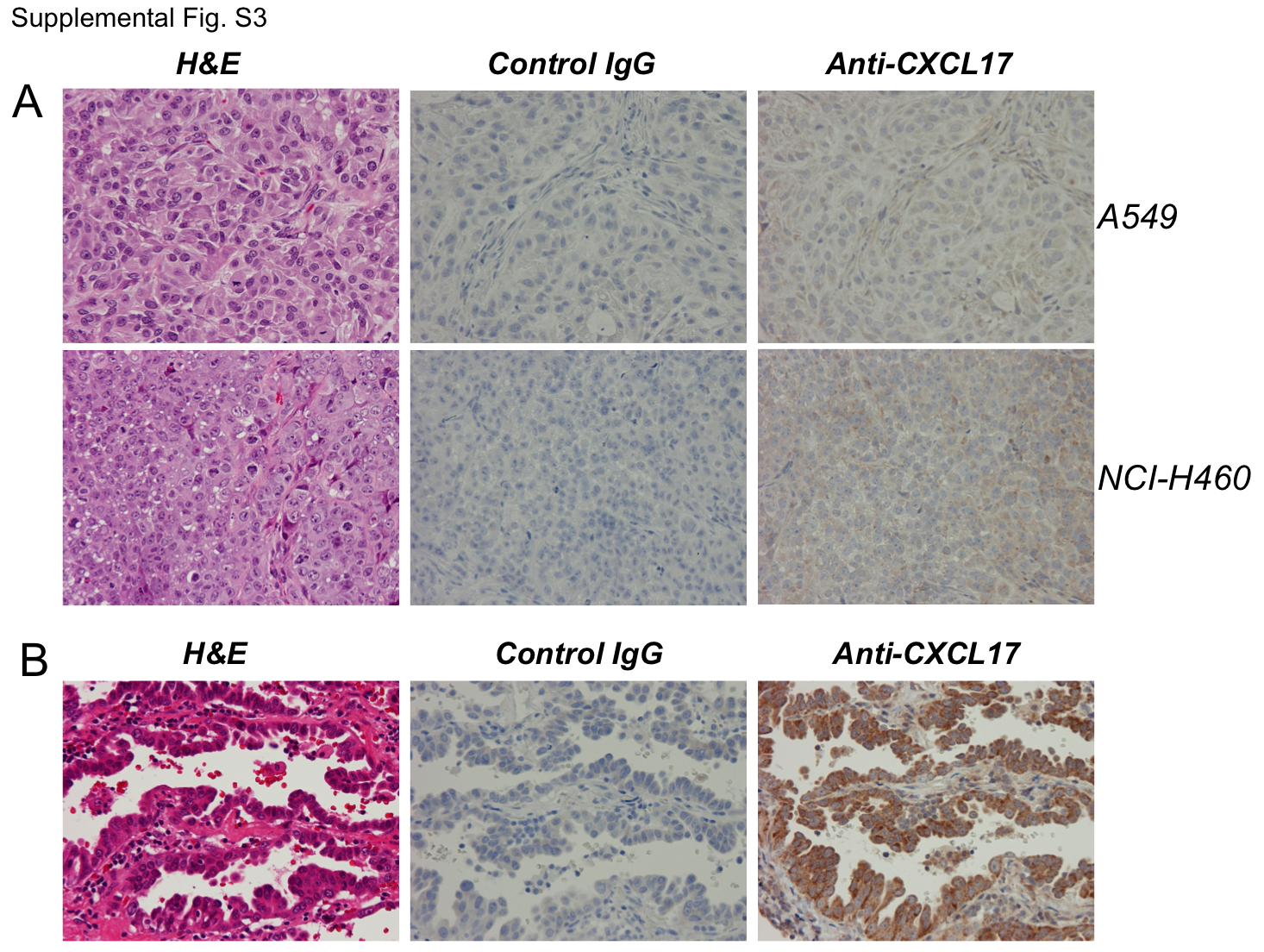

Supplement: Figure S3 — Immunohistochemistry for CXCL17 in the xenograft tumor and in surgically resected specimens from human lung adenocarcinoma. (A) Immunohistochemistry for CXCL17 in the xenograft tumor of A549 and NCI-H460 cells. (B) Surgically resected lung cancers were probed with anti-human CXCL17 antibodies. Magnification is x 100. The brown color (3,3′-diaminobenzidine) indicates positive staining. Left panels, H&E staining; middle panels, staining with normal rabbit IgG; right panels, anti-human CXCL17 IgG. (TIF) [file pone.0044080.s003.tif]

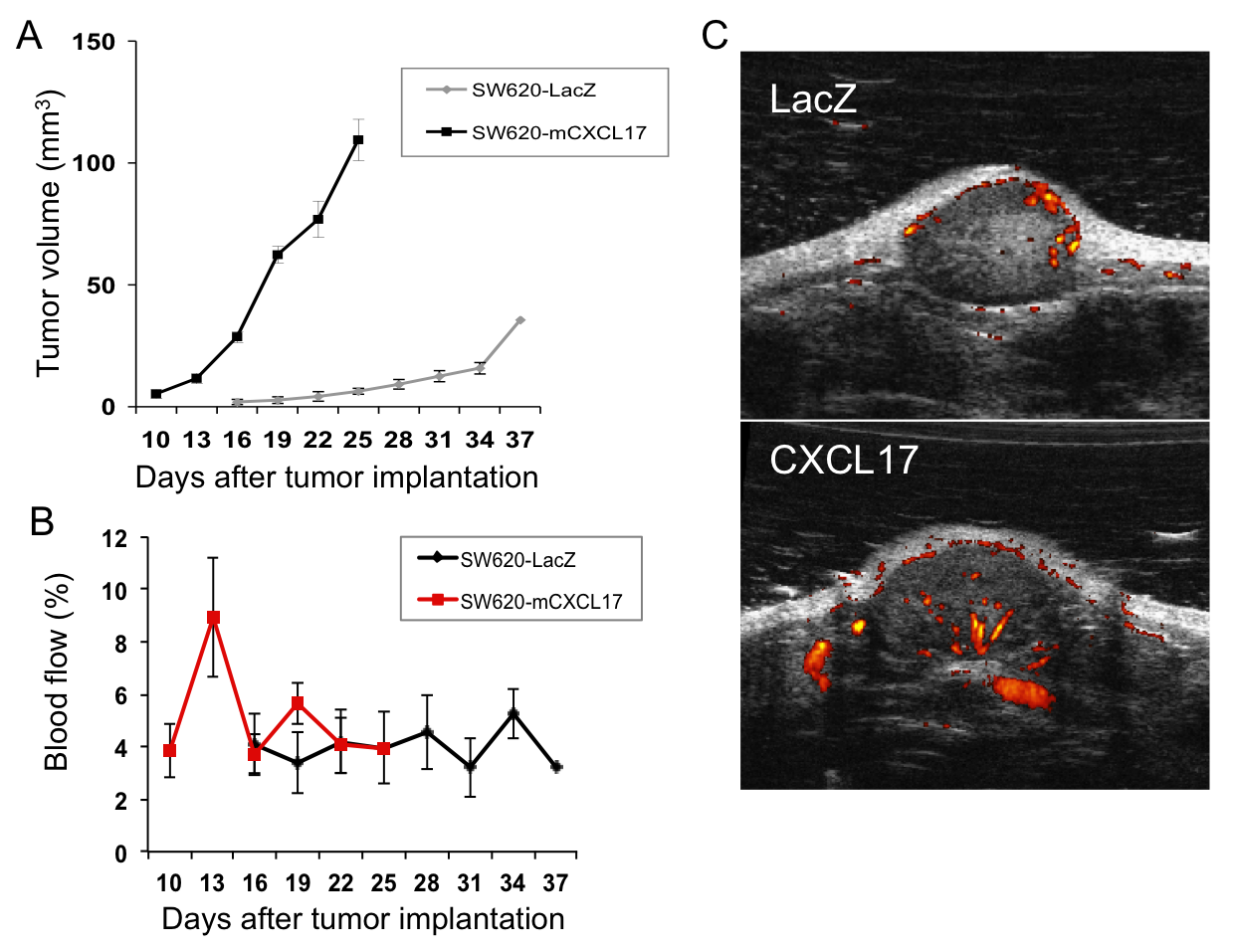

Supplement: Figure S4 — Enhanced tumor formation in CXCL17-expressing SW620 colon cancer cells. (A) CXCL17-transfected SW620 cells (1×10^6^) were transplanted into the subcutaneous space of C.B-17 SCID mice and tumor volume was measured at the indicated time points by Vevo770. (B) Blood flow analysis using Vevo770 Doppler-based ultrasound imaging analysis in CXCL17-SW620 cells. It is worth noting that tumors of CXCL17-expressing SW620 cells showed increased blood flow signals in the early tumorigenic stage, but not those of control LacZ-SW620 cells. (C) Stronger blood flow signals in CXCL17-SW620 tumors compared to LacZ-SW620 tumors at the equivalent tumor volume (75 mm3). (TIF) [file pone.0044080.s004.tif]

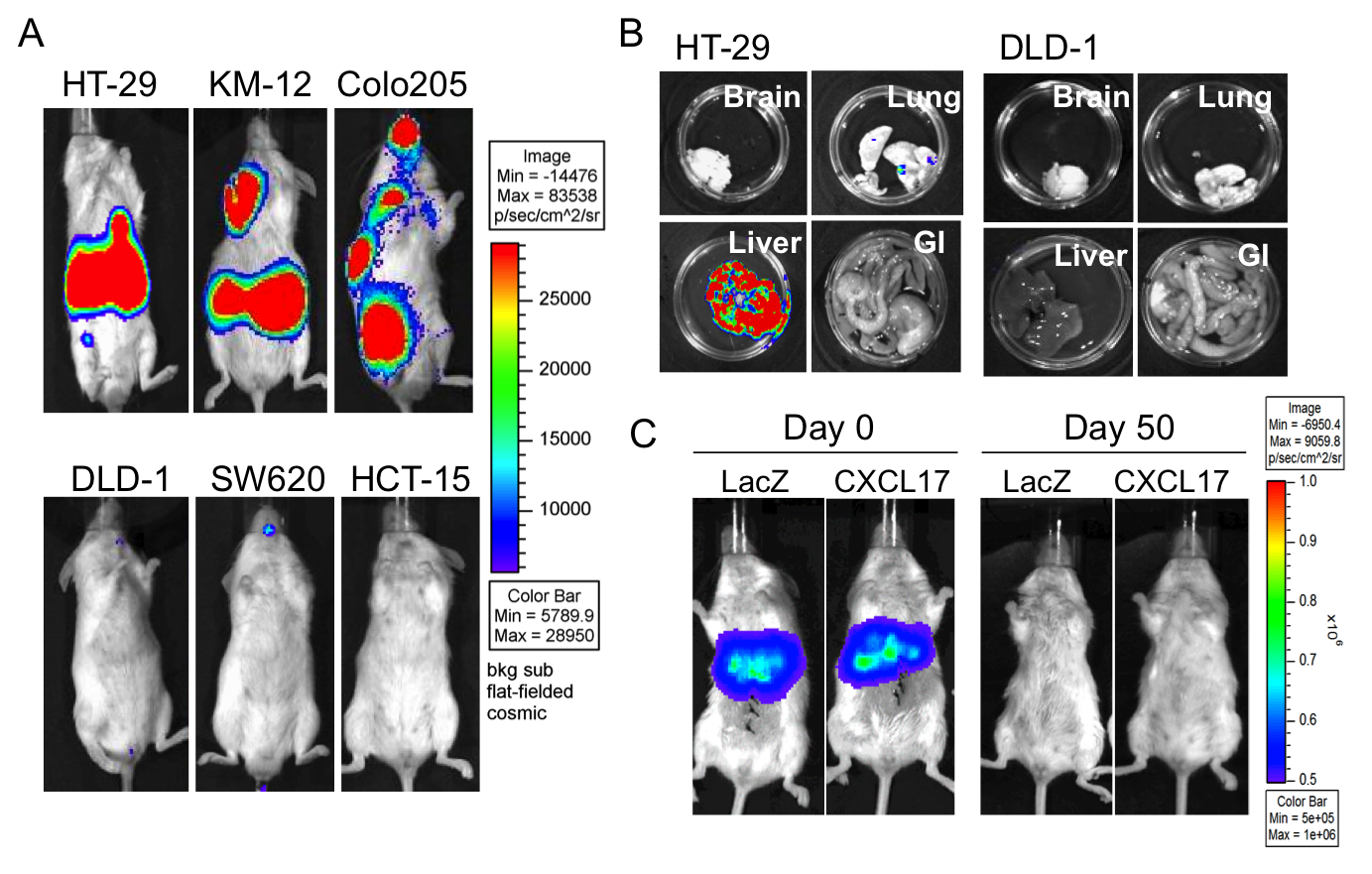

Supplement: Figure S5 — Metastatic potential in CXCL17-expressing human cancer cell lines. (A) Luciferase-expressing colon cancer cells were injected into the left ventricle of NOD/SCID mice. Metastatic growth was monitored by tumor-derived photons in vivo. Representative images around 30–40 days following tumor injection are shown. It is worth noting that DLD-1, SW620 and HCT-15 cells were less metastasized than HT-29, KM-12 and Colo205 cells. (B) Ex vivo luminescent inspection of metastasized organs after intra-cardiac injection of tumor cells. (C) Luciferase and CXCL17 doubly expressing DLD-1 cells were injected into the portal vein of SCID mice and then examined by in vivo luminescent imaging. (TIF) [file pone.0044080.s005.tif]

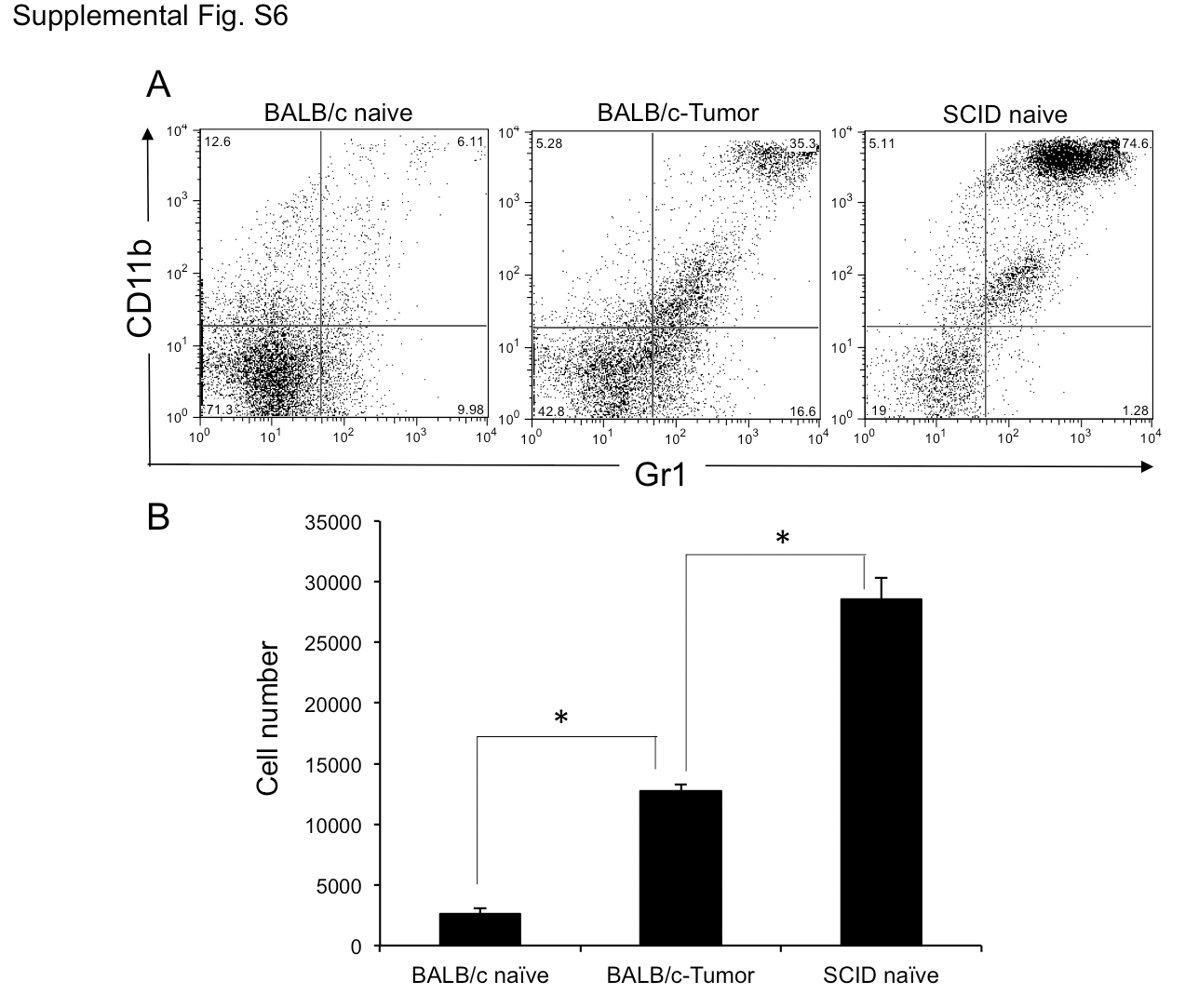

Supplement: Figure S6 — Increase in CXCL17-responding cells in tumor-bearing BALB/c mice. Parental (unmanipulated) Colon26 cells were transplanted subcutaneously into BALB/c mice, and tumor-bearing conditions in BALB/c mice conditions were generated (BALB/c Tumor, at 15–20 days after implantation). (A) Spleen cells were isolated from the indicated mice and the number of CD11b+Gr-1+ cells was determined by a flow cytometer. It is interesting to note that CD11b+Gr-1+ cells in BALB/c Tumor increased more than in naïve tumor-free mice. (B) Spleen cells were isolated from the indicated mice and used for the chemotaxis assay using mouse recombinant CXCL17. *, P<0.001 (n = 4). One of two independent experiments with similar results is shown. (TIF) [file pone.0044080.s006.tif]

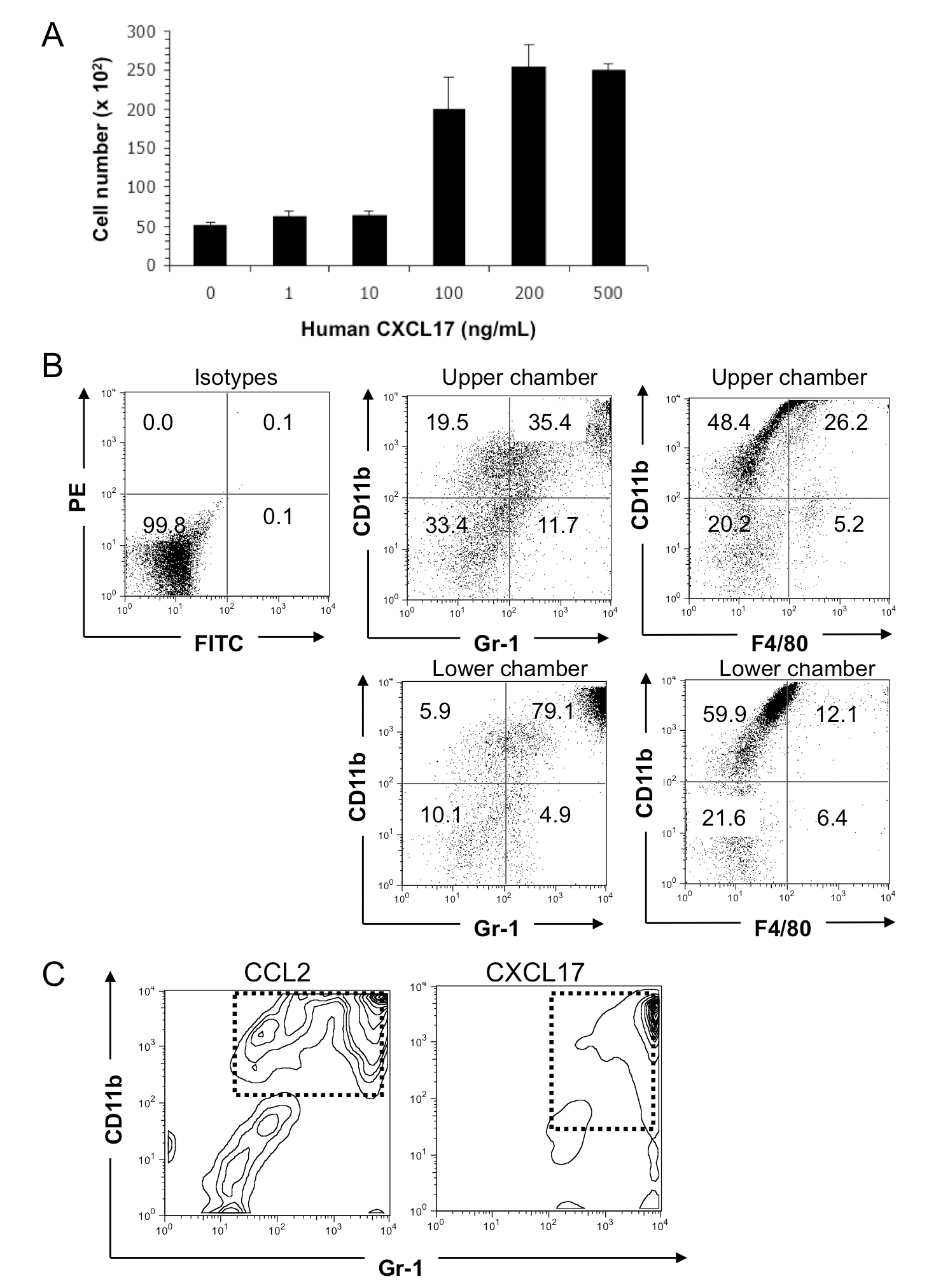

Supplement: Figure S7 — Flow cytometric analysis of CXCL17-responding cells. (A) Spleen cells were isolated from SCID mice and used for the chemotaxis assay using human recombinant CXCL17. (B) Human recombinant CXCL17-responding cells were stained with PE-conjugated anti-mouse CD11b, FITC-conjugated anti-mouse F4/80 and FITC-conjugated anti-mouse Gr-1 mAbs, and then analyzed using flow cytometry. Isotypes, staining with PE-conjugated and/or FITC-conjugated isotype-matched control IgGs; Upper chamber, remaining cell population; lower chamber, responding cell population. (C) Differential population of CXCL17-responding cells from CCL2-responding cells. The dotted rectangle represents the major population preferentially responding to the indicated chemokine. (TIF) [file pone.0044080.s007.tif]

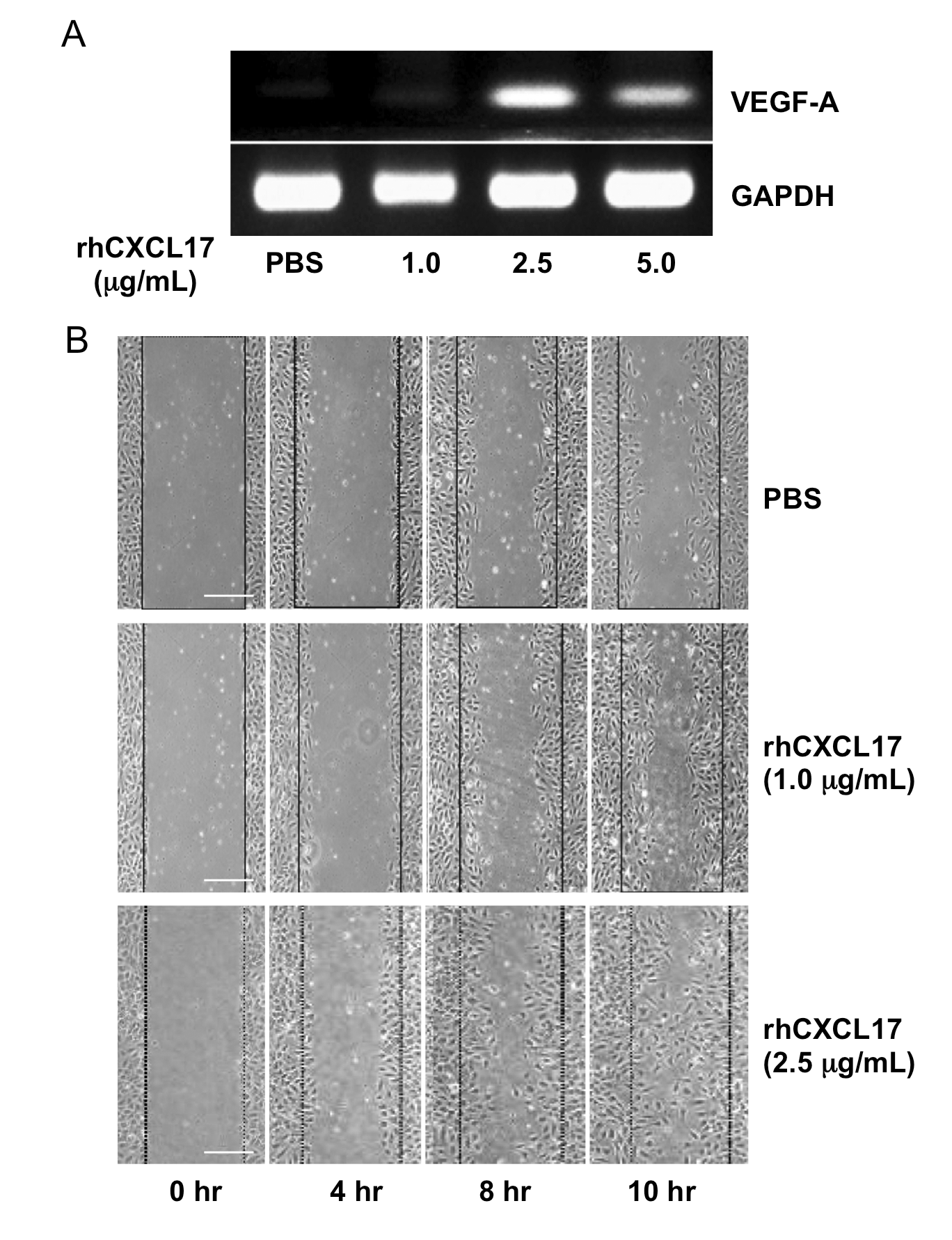

Supplement: Figure S8 — Enhanced migration of HUVECs with CXCL17. (A) RT-PCR analysis of VEGF-A mRNA expression in HUVECs following recombinant hCXCL17 treatment. HUVECs were exposed to CXCL17 at the indicated concentrations for 10 hr. Upper panel, VEGF-A; lower panel, GAPDH as an internal control. (B) Migration assay of HUVECs in the presence of recombinant hCXCL17. Lines represent reference points for the scratch with a pipette tip. One of two independent experiments with similar results is shown. (TIF) [file pone.0044080.s008.tif]
