# Supplementary material for: CXCL17 Expression by Tumor Cells Recruits CD11b+Gr1highF4/80− Cells and Promotes Tumor Progression
Source: PLoS One. 2012 Aug 29;7(8):e44080. doi: 10.1371/journal.pone.0044080 (PMC3430639; doi:10.1371/journal.pone.0044080)
Supplement: Materials and Methods S1 — (DOC) [file pone.0044080.s009.doc]

**Supporting Materials and Methods**

##### ***In vivo luciferase imaging***

1. Metastatic tumor progression was monitored using the non-invasive bioimaging system IVIS^TM^ (Xenogen, Alameda, CA) [34, 36]. For a systemic metastatic model, luciferase-expressing cell lines were injected into the left ventricle of mice under ultrasonic guidance (Vevo770, VisualSonics). For tumor implantation to the liver, a midline incision was made on SCID mice anesthetized with isoflurane (Abbott Laboratories, North Chicago, IL), and luciferase/mCXCL17-expressing DLD-1 and SW620 cells (2 X10^5^) in 0.2 ml of PBS were injected into the ileocolic vein [13]. After tumor-implanted mice were anesthetized with a 2%/98% isoflurane/oxygen mixture, and D-luciferin (potassium salt; Biosynth, Postfach, Switzerland) was injected into the peritoneal cavity at 2 mg/body, measurement was then immediately made of luciferase activity. After acquiring photographic images of each mouse, luminescent images were obtained with an exposure time of 1~15 min [13, 34, 35]. Optical images were displayed and analyzed using Igor (WaveMetrics, Lake Oswego, OR) and IVIS Living Image (Xenogen) software packages. The signal from tumors was quantified as photons flux in units of photons/sec/cm^2^/steradian.

***Migration assay for vascular endothelial cells***

Human umbilical vein endothelial cells (HUVECs) were purchased from Cascade Biologics (Portland, OR) and maintained in EBM-2 **®** Endothelial Basal Medium with supplement (Lonza, Walkersville, MD) in accordance with the manufacturer’s instructions. HUVECs were plated onto the prepared 60-mm dish to create a confluent monolayer. The cell monolayer was scraped in a straight line to create a "scratch" with a pipette tip (P1000). To obtain the same field during the image acquisition, markings were created as reference points close to the scratch. The recombinant proteins were added at the indicated concentrations. The culture dishes were placed in a tissue culture incubator at 37°C for 0–10 hr. The dishes were taken out of the incubator for periodical examination (photographed images) and then returned to resume incubation until the next inspection.
